# Relative Bioavailability of Niacin Supplements for Dairy Cows: Effects of Rumen Protection and of Feed Processing

**DOI:** 10.3390/vetsci2040440

**Published:** 2015-12-16

**Authors:** Reka Tienken, Susanne Kersten, Liane Hüther, Jana Frahm, Ulrich Meyer, Sven Dänicke

**Affiliations:** Institute of Animal Nutrition, Friedrich-Loeffler-Institute (FLI), Federal Research Institute for Animal Health, Bundesallee 50, Brunswick 38116, Germany; E-Mails: reka.tienken@fli.bund.de (R.T.); susanne.kersten@fli.bund.de (S.K.); liane.huether@fli.bund.de (L.H.); jana.frahm@fli.bund.de (J.F.); sven.daenicke@fli.bund.de (S.D.)

**Keywords:** dairy cow, niacin, bioavailability, feed processing, pharmacokinetic

## Abstract

The present study aimed to examine the effective systemic bioavailability of niacin— with particular focus on its galenic form—and feed processing. Experiment 1 was conducted with 35 dairy cows to investigate the effects of various doses of oral supplemented nicotinic acid (NA) either in differing galenic forms (non-rumen protected (nRP) *vs.* rumen protected form (RP)) on serum niacin concentrations. Experiment 2 was designed as a pharmacokinetic study examining the serum niacin kinetics over 24 h after giving a single oral bolus of 24 g nRP or RP NA admixed in either pelleted or ground concentrate. In both experiments, only the niacin vitamer nicotinamide (NAM) was detected. Results of experiment 1 showed that both galenic forms at a dose of 24 g/cow daily elevated NAM concentrations at the beginning of the experiment. Despite a daily supplementation, NAM concentrations decreased continuously towards the end of the experiment which was more steeply in nRP NA (*p* = 0.03). On experimental day 21, NAM concentrations were higher when feeding RP NA (*p* = 0.03) and the highest dose (24 g/day and cow) (*p* < 0.01). Results of experiment 2 indicated that nRP and RP were characterized by similar pharmacokinetic profiles resulting in similar areas under the curves as a net result of the kinetic counterbalancing alterations. Pelleting seemed not to influence the relative bioavailability.

## 1. Introduction

Niacin (vitamin B_3_) and its vitamers nicotinamide (NAM) and nicotinic acid (NA) are important for the synthesis of the coenzymes NAD and NADP which are involved in a large number of biological pathways [1]. The NRC [2] declared the requirement of niacin for a lactating cow with a body weight of 650 kg and 35 kg milk yield with 289 mg per day and it was shown that ruminants are capable of covering these requirements from different sources. On the one hand, feedstuffs containing niacin and the synthesis from tryptophan and quinolinic acid via salvage and *de novo* pathway provide one source for covering niacin requirements [1,3]. On the other hand, Santschi, *et al.* [4] determined a ruminal niacin synthesis of 2213 mg niacin per day in multiparous and fistulated Holstein cows (between 29 and 178 days in milk (DIM); milk production 27.7 ± 1.4 kg/d).

Niacin and its vitamers have recently attracted increased interest as feed additives due to their promising targets for diverse effects on bovine metabolism. It was shown that feeding additional niacin as nicotinic acid (6–12 g niacin/d) influenced the ruminal ecosystem like the stimulation of the microbial protein synthesis [5], the increase of total protozoal counts [6,7] and the increase of butyric acid, valeric acid, propionic acid, and ammonia [5,6,7,8]. It was stated that the supplementation with NA increase the milk yield [9] and induce shifts in the milk composition [10,11,12]. Furthermore, NA as feed additive was suggested to balance catabolic metabolism postpartum, because it was shown that NA is able to stimulate GPR109A receptor resulting in a down-regulation of the lipolysis via the dephosphorylation of the hormone-sensitive lipase in adipose tissue [13].

Santschi, *et al.* [4] stated that non-rumen protected (nRP) niacin is massively degraded within the rumen. To improve rumen stability of supplemented niacin and therefore increase duodenal bioavailability, new vitamin encapsulation techniques were developed. However, results of studies investigating the effects of rumen protected (RP) NA on bovine metabolism and production were also highly variable [14,15] rising the question whether the rumen protection results in an increased or compromised systemic niacin bioavailability.

Because there are no studies comparing the pharmacokinetics of nRP and RP, the present study was conducted employing a feeding experiment (Experiment 1) and a pharmacokinetic approach (Experiment 2) in order to assess the systemic niacin bioavailability. Moreover, as concentrate feed is often used in a pelleted form for technical reasons, this feed processing step was additionally addressed in Experiment 2.

## 2. Experimental Section

In accordance with the German Animal Welfare Act, pertaining to the protection of experimental animals and approved by the Lower Saxony State Office for Consumer Protection and Food Safety (LAVES), Oldenburg, Germany, two experiments were carried out at the experimental station of the Institute of Animal Nutrition, Friedrich-Loeffler-Institute (FLI), Brunswick, Germany.

The basal diet of both experiments consisted of 30% concentrate and 70% roughage mixture on dry matter (DM) basis and was provided as partial mixed ration (PMR). The roughage mixture was composed of 60% maize silage and 40% grass silage and cows were fed at 5:30 a.m. and 3:00 p.m. daily.

The nRP (Niacin feed grade, Lonza Ltd., Basel, Switzerland) and the RP NA (Niashure rumen protected niacin, Balchem Corporation, New Hampton, NY, USA) were included into the pelletized or ground concentrate compromising 12 g NA/kg. For this reason 12.1 g nRP and 18.5 g RP NA were added per kg concentrate.

### 2.1. Animals and Treatments in Experiment 1

Thirty-five late-lactating dairy cows (241 ± 25 DIM) were distributed to one of seven dietary treatment groups. The experiment lasted 21 days and the animals were allocated with regard to lactation number (2.9 ± 0.1), milk yield (27 ± 1 kg/d), and body weight (653 ± 21 kg) resulting in nearly homogeneous groups of five animals each. The cows were kept in a free stall barn with slatted floors and cubicles covered with rubber mats. The factors studied were: (1) the dose of the NA supplement (6, 12, or 24 g NA/d and cow) and (2) the galenic form of the NA supplement (non-rumen protected (nRP) *vs.* rumen protected (RP)). Treatment groups of the experiment were: no additive (CON); daily supplementation of 6 g nRP NA (6_nRP); 12 g nRP NA (12_nRP); 24 g nRP NA (24_nRP); 6 g RP NA (6_RP); 12 g RP NA (12_RP); 24 g RP NA (24_RP). Therefore, a complete 3 × 2 factorial design with three levels of NA (6, 12, and 24 g/cow and d) and two galenic forms of the NA supplement (nRP, RP) was tested. The additional CON group was tested to record the general performance level without any NA supplement.

The PMR was provided in self-feeding stations (TYPE RIC, Insentec, B.V., Marknesse, The Netherlands). The dietary treatment was provided by a computerized concentrate feeding station (Insentec, B.V., Marknese, The Netherlands). The niacin components were included into the pelletized concentrate to provide daily doses of 6, 12, 24 g corresponding to 0.5, 1, or 2 kg. The difference to the total amount of 2 kg was completed with CON concentrate. Thus, cows without NA supplementation received 2 kg of CON.

### 2.2. Animals and Treatments in Experiment 2

The present study was conducted in a 2 × 2 factorial design with four late-lactating cows (241 ± 32 DIM). The cows were fitted with a venous catheter in the *Vena jugularis externa* and had an average body weight of 544 ± 62 kg. The lactation number ranged from the first to the fifth lactation and the average milk yield was 25 ± 5 kg/d. Cows were kept in a tethered stall with neck straps. Each cow had its own trough, free access to water, and to a salt block containing sodium chloride throughout the experiment. Cows were given two weeks for adaption to housing and feeding regimen which was followed by 16 days of sampling which was divided into five periods. The interval between each period was at least 72 h and maximum 96 h.

The factors studied were: (1) the galenic form of the NA supplement (nRP *vs.* RP) and (2) the processing form of the dietary concentrate (pellet (P) *vs.* meal (M)).

Each cow received each dietary treatment once at the beginning of each period (5:30 a.m.). The dietary treatment was provided before the feeding of the PMR to ensure a complete uptake. Cows of the present experiment received 2 kg of nRP_P, nRP_M, RP_P and RP_M to ensure an uptake of 24 g NA. In the following days of each period, cows received a control ration with CON instead of a dietary treatment. Treatments of the experiment were: no additive (CON); bolus of 24 g nRP NA in pelleted concentrate (nRP_P); bolus of 24 g nRP NA in ground concentrate (nRP_M); bolus of 24 g RP NA in pelleted concentrate (RP_P); bolus of 24 g RP NA in ground concentrate (RP_M).

### 2.3. Data Collection and Analysis

The concentrate intake of the animals was recognized individually via an ear transponder, whereas the intake of the PMR was recorded per group in experiment 1. In experiment 2, feed refusals of the individual feed toughs were removed and weighted daily.

Cows of both experiments were milked twice daily at 5:30 a.m. and 3:30 p.m. The body weight was detected automatically after each milking when leaving the milking parlour in experiment 1. In experiment 2, cows were weighed at the beginning and the end of the experiment. Milk samples of experiment 1 were collected twice a week during the morning and afternoon milking and were fixed with Bronopol and stored at 8 °C until analysis. The milk samples were analyzed for fat, protein, and lactose using a milk analyzer based on Fourier transform infrared spectroscopy (Milkoscan FT 6000, Foss Electric, Hillerød, Denmark) and combined with a flow cytometric measurement for somatic cell count analysis (Fossomatic 500, Foss Electric, Hillerød, Denmark).

Samples of roughages were taken two times weekly, whereas samples of concentrates were taken once a week. Samples were pooled over approximately four weeks. Feed samples were dried at 60 °C for 72 h and ground before analysis. Dry matter, crude ash, crude fiber, crude protein, ether extract, neutral detergent fiber, and acid detergent fiber in maize and grass silage and concentrates were analyzed according to the methods of the Association of German Agricultural Analytic and Research Institutes (VDLUFA) [16]. To ensure the intended concentrate to forage ratio, the DM of the forages were determined twice a week in both experiments. Table 1 shows the chemical composition of the experimental feedstuffs. 

**Table 1:** Components, chemical composition, and content of nicotinic acid (NA) of concentrates and roughages offered in the present experiments. The galenic forms non-rumen protected (nRP) and rumen protected (RP) NA were admixed either in pelleted (P) or ground (M) concentrate.

	Concentrates						Roughages	
	CON ^1^	nRP ^2^		RP ^3^		C-lak ^4^	Maize Silage	Grass Silage
	P ^5^	P ^5^	M ^6^	P ^5^	M ^6^	P ^5^		
Components (% of the original substance)								
Soy extracted meal	26.0	26.0	26.0	26.0	26.0	26.0		
Wheat	50.0	48.8	48.8	48.2	48.2	50.0		
Maize	20.0	20.0	20.0	20.0	20.0	20.0		
Mineral premix ^7^	4.0	4.0	4.0	4.0	4.0	4.0		
nRP NA ^8^	-	1.2	1.2	-	-	-		
RP NA ^9^	-	-	-	1.9	1.9	-		
Chemical composition								
Dry matter (g/kg)	884	885	887	889	890	884	359	300
Nutrient (g/kg dry matter)								
Crude ash	63	63	72	64	68	63	41	104
Crude protein	220	227	238	217	226	212	93	138
Ether extract	28	28	26	33	32	29	29	35
Crude fiber	43	37	35	37	36	37	175	294
NDF	157	180	149	167	154	202	380	519
ADF	45	51	49	50	49	47	199	311
Energy ^10^ (MJ/kg dry matter)								
NE_L_	8.3	8.5	8.3	8.5	8.3	8.3	6.5	6.6
NA ^11^ (g/kg dry matter)	0.05	12	12	12	12	0.05	0.05	0.03

**^1^** CON, control concentrate. **^2^** nRP, non-rumen protected nicotinic acid; **^3^** RP, rumen protected nicotinic acid. **^4^** C-lak, concentrate included in the partial mixed ration; **^5^** P, pelleted concentrate; **^6^** M, ground concentrate; **^7^** Mineral premix, per kilogram: 140 g Ca, 120 g Na, 70 g P, 40 g Mg, 6000 mg Zn, 5400 mg Mn, 1000 mg Cu, 100 mg I, 40 mg Se, 25 mg Co, 1,000,000 IU vitamin A, 100,000 IU vitamin D3, 1500 mg vitamin E; **^8^** nRP NA, non-rumen protected nicotinic acid; **^9^** RP NA, rumen protected nicotinic acid; **^10^** Calculation based on nutrient digestibilities measured with wethers [17]; **^11^** NA, nicotinic acid. The contents of the non-rumen protected and the rumen protected NA based on manufacturer information. The contents of nicotinic acid of the roughages and the remaining concentrates based on table values listed in Ballet, *et al.* [18] and NRC [2].

Blood sampling started at 7:30 a.m. and cows were captured in a self-locking fence in experiment 1. Blood was taken out of the *Vena jugularis externa*, on experimental day 0, 1, 3, 6, 10, and 21 of the groups 24_nRP and 24_RP, while those of the other groups were only taken on day 21.

Blood was taken out of the catheterized *Vena jugularis externa* in experiment 2. The zero-blood sample of each period was collected at 5:00 a.m., 30 min before the morning feeding. Further blood samples were taken 60, 120, 180, 240, 300, 360, 480, 600, 720, and 1440 min after zero-blood sampling.

#### 2.3.1. Dietary Niacin Content

The content of niacin in the feedstuffs were calculated based on table values [2,18] and on the information of the niacin manufacturers.

#### 2.3.2. Blood Niacin Concentrations

Blood samples of both experiments were centrifuged at 3000× *g* for 30 min at 15 °C and the serum obtained was stored at −80 °C until analysis. Serum samples were analyzed for the concentrations of NAM and NA using HPLC. At first, samples were thawed at 20 °C and then homogenized which was followed by protein precipitation and fat extraction using ice cold ethanol and *n*-hexan. After centrifugation at 14,000 rpm, the supernatant was transferred into an amber flask and evaporated in a nitrogen stream at 40 °C and the residue was dissolved in 150 µL of the aqueous mobile phase A. After filtration (syringe filters, 0.45 µm, PVDF, amchro GmbH) 20 µL of the filtrate were injected automatically into a HPLC system (model SCL-10A controller, model LC-10AS pump, model SIL-10AC autosampler, model CTO-10AC oven; Shimadzu, Kyoto, Japan). Samples were run through a C18 column (Insertil ODS, 150 × 3 mm, 5 µm particle size, 150 Å pore size) by using a binary gradient system at a flow rate of 0.4 mL/min. The composition of mobile phase A was 10 mM sodium 1-hexanesulfonate monohydrate in ultrapure water at a pH of 2.3, while mobile phase B was 100% acetonitrile. The gradient profile started with 100% mobile phase A for 10 min, followed by a linear decline to 97% during 10 min and to 60% mobile phase A within the next 2 min. During the following 10 min the system returned to the initial conditions. Quantification of NAM and NA was done simultaneously by a multi wavelength detector at a wavelength of 260 nm. The retention times of NA and NAM were 13.5 min and 18.7 min, respectively. For preparation of standard solutions NA and NAM from Sigma-Aldrich (Steinheim, Germany) was used. Stock solutions (400 µg·mL^−1^) were prepared in bi-distillated water and stored at −20 °C. Mixed standard working solutions in the range of 0.20–2.00 µg·mL^−1^ were obtained by dilution of stock solutions with mobile phase A directly before use. Quantification was performed comparing peak area with standard curves.

### 2.4. Calculations and Statistics

Fat-corrected milk (FCM) was calculated following Gaines [19]:

(1) FCM (kg/d) = (( milk fat ⋅ 0.15) + 0.4)  kg milk yield

#### 2.4.1. Experiment 1

For analyzing the performance parameters PROC MIXED procedure of the SAS-software package (SAS Enterprise Guide 6.1) was used. Data were analyzed in two steps. First, all experimental groups were considered as separate treatments irrespective of further possible classification traits resulting in a one-factorial design with the treatment group (*N* = 7) as fixed factor. Secondly, the CON group was excluded from data evaluation to enable the more powerful examination of the pooled effects of NA dose (6, 12 or 24 g) and of the galenic form of the NA supplements (nRP or RP) resulting in a complete 3 by 2 two-factorial design with NA dose, galenic form and their interactions as fixed factors.

For both evaluation strategies, the frequent time-dependent measurements during the experiment for each individual cow were considered as repeated measures where applicable (24_RP; 24_nRP). After testing various structures for the model, the first order autoregressive (ar(1)) showed the lowest Akaike information criterion.

For assessing the serum niacin concentrations of 6_nRP, 12_nRP, and 24_nRP as well as 6_RP, 12_RP, and 24_RP on day 21, the PROC MIXED procedure of the SAS-software package (SAS Enterprise Guide 6.1) was used and the dose (6, 12, or 24 g) and the galenic form of the NA supplement (nRP or RP) and the interaction between those factors were set as fixed effects.

For time-sequence data of 24_nRP and 24_RP, linear regression analysis and subsequent slope comparisons were performed using the PROC MIXED procedure of the SAS-software package (SAS Enterprise Guide 6.1) to investigate the time-depending blood profiles between 24_nRP and 24_RP during the three week feeding period. In particular, the model included the galenic form as fixed factor and the experimental day as a covariate with the galenic form nested in the covariate to enable estimating linear regression coefficients (slopes) for both galenic forms which characterize the blood NAM changes over time. The individual animal was handled as a random factor to consider similarity within individuals. The difference between both regression slopes was tested for significance using the “ESTIMATE” statement.

#### 2.4.2. Experiment 2

For evaluating the data a non-linear regression model of the application STATISTICA (StatSoft, version 10) was used to fit the data of nRP_P, nRP_M, RP_P, and RP_M according to Mercer, *et al.* [20]:

(2) $$\text{Niacin }\left(\frac{µ\text{g}}{\text{mL}}\right)=\frac{{\left(\text{B}\cdot \text{K}0.5^{\text{n}}\;+\text{ Rmax}\cdot {\text{x}}^{\text{n}}+\text{B x }\begin{array}{c} \left(2\text{ n}\right)/{\text{Ks}}^{\text{n}}) \end{array}\right)}_{\;}^{\;}}{{\left(\text{K}0.5^{\text{n}}+{\text{x}}^{\text{n}}+{\text{x}}^{\left(2\text{ n}\right)/{\text{Ks}}^{\text{n}}}\right)}^{\;}\;}$$

where *R*_max_ is the maximum theoretical NAM concentration, x is the apparent kinetic order, *K*_0.5_ is the time for ½ of *R*_max_ and *K*_s_, is the time indicative for the decreasing part of the regression. The estimated parameters were used to calculate the area under the serum concentration time curve (AUC) numerically by applying the trapezoidal method with the formula:

(3) AUC=∑i = 2n Concentration i (Δ time i=1 +  (Δ time i ))2

with ∆time_n_ = 0.

The parameter *I*_max_, the time of occurrence of *R*_max_, was calculated as follows:

(4) I max=(K s   K 0.5)0.5

As feeding of the basal (CON) concentrate feed did not reveal a distinct systemic niacin peak the corresponding time-dependent blood concentrations were not fitted to Equation (2) but just subjected to AUC determination according to Equation (3).

The PROC “MIXED” procedure of the SAS was used evaluating the pharmacokinetic parameters of the serum samples. The form of the concentrate (pellet or meal) and the galenic form of the NA supplement (nRP or RP) and the interaction between those factors were considered as fixed effects. The cow which is the subject was considered as a random effect.

Before calculating the relative bioavailability with the following formula, the control feeding related AUC was subtracted from the corresponding AUCs of the other dietary treatments.

(5) $$\text{Relative bioavailability}=\frac{AUC_{24}\text{ dietary treatment }}{AUC_{24}\text{ reference treatment }\left({\text{nRP}}_{-}\text{M}\right)}$$

with non-rumen protected NA mixed into the concentrate and presented in a meal form (nRP_M) declared as the reference treatment.

In all statistical investigations using PROC MIXED procedure of SAS, degrees of freedom of PROC MIXED procedure were calculated using the Kenward-Roger method. For determination of differences between LSMeans, the probability (“PDIFF”) option was used applying a Tukey-Kramer test for post-hoc analysis. Differences were considered to be significant when F-test statistics revealed *p* < 0.05, whereas a tendency was noted if *p* < 0.10 and *p* > 0.05.

## 3. Results

Only concentrations of NAM were detected in serum samples while the concentrations of NA was always lower than the detection limit.

### 3.1. Experiment 1

The overall performance level of the cows is reflected by the pooled average daily DMI of 20.3 kg at the first experimental day, and 18.9 kg on day 21 of the experiment. The overall data evaluation revealed no significant differences between the treatment groups and the CON group (The mean milk yield and FCM of group CON were 25.7 ± 4.2 kg/d and 28.8 ± 4.8 kg/d. Mean yield of milk fat, milk protein, and milk lactose were 1.2 ± 0.2 kg/d, 0.90 ± 0.11 kg/d, 1.2 ± 0.2 kg/d, whereas mean urea concentration was 203 ± 36.8 ppm.).

Excluding the CON group from data evaluation enabled a more powerful examination of the pooled variance caused by NA dose and galenic form, *i.e.*, by evaluating the data according to a complete 3 (NA doses) by 2 (galenic forms) design (Table 2). However, even under these data evaluation conditions no significant effects of NA dose or galenic form could be identified for the performance parameters.

**Table 2:** Effects of oral supplementation of 6, 12, or 24 g non-rumen protected or rumen protected nicotinic acid on lactation performance of dairy cows (Experiment 1).

Lactation Performance	Dietary Treatment							*p*-Value		
	6_nRP ^1^	12_nRP ^2^	24_nRP ^3^	6_RP ^4^	12_RP ^5^	24_RP ^6^	PSEM ^7^	F ^8^	D ^9^	F × D
Milk yield (kg/d)	27.7	25.5	25.1	25.3	25.8	27.7	26.2	0.91	0.85	0.34
Milk fat (kg/d)	1.3	1.2	1.1	1.2	1.0	1.3	1.2	0.63	0.24	0.26
FCM ^**10**^ (kg/d)	30.6	27.4	27.2	29.9	26.3	29.5	28.5	0.93	0.37	0.73
Milk protein (kg/d)	0.96	0.93	0.85	0.92	0.90	0.96	0.92	0.83	0.78	0.27
Milk lactose (kg/d)	1.3	1.2	1.2	1.2	1.2	1.3	1.3	0.71	0.71	0.35
Milk urea (ppm)	191	188	183	189	179	189	187	0.82	0.79	0.76

**^1^** 6_nRP, cows received 6 g non-rumen protected (nRP) nicotinic acid (NA) per day. **^2^** 12_nRP, cows received 12 g nRP NA per day; **^3^** 24_nRP, cows received 24 g nRP NA per day; **^4^** 6_RP, cows received 6 g RP NA per day; **^5^** 12_RP, cows received 12 g RP NA per day; **^6^** 24_RP, cows received 24 g RP NA per day; **^7^** PSEM, pooled standard error of mean. **^8^** Form, non-rumen protected (nRP) or rumen protected (RP) nicotinic acid; **^9^** Dose, 6 g (6), 12 g (12) or 24 g (24) NA per day; **^10^** FCM, fat-corrected milk yield.

**Figure 1:**
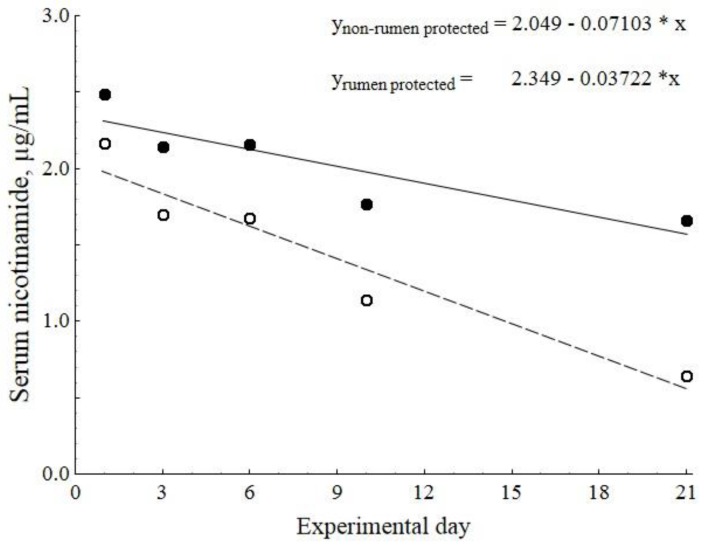
Linear regression of the serum levels of nicotinamide during three weeks of feeding 24 g/d non-rumen protected (dashed line with open circles) or rumen protected (solid line with filled circles) nicotinic acid (NA) to dairy cows (Experiment 1). The slopes of the linear regressions were significantly different (*p* = 0.03).

The NAM concentrations of 24_nRP and 24_RP were investigated on day 1, 3, 6, 10, and 21. As shown in Figure 1, the serum NAM concentration decreased linearly after the first NA uptake in both groups which was more steeply in 24_nRP compared to 24_RP (*p* = 0.03).

**Figure 2:**
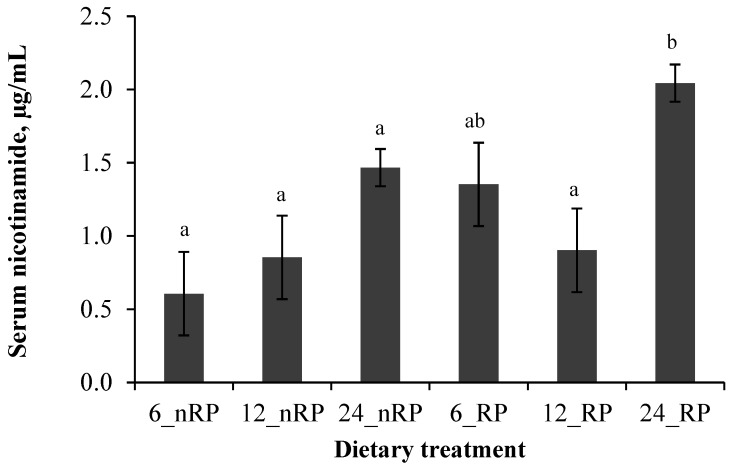
Serum nicotinamide concentrations of dairy cows following oral supplementation of 6, 12, or 24 g/d non-rumen protected (nRP) or rumen protected (RP) nicotinic acid for three weeks (Experiment 1). The serum NAM concentrations were influenced by the galenic form of the supplement (*p* = 0.03) and the dose of the supplement (*p* < 0.01), whereas the interaction between the galenic form and dose remained unaffected (*p* = 0.41).

As presented in Figure 2, the serum NAM concentrations were higher in RP_NA compared to nRP_NA (*p* = 0.03) and 24 g of NA resulted in the highest NAM concentrations (1.76 ± 0.09) followed by 6 g (0.98 ± 0.20) and 12 g (0.88 ± 0.20) NA (*p* < 0.01) (Figure 2).

### 3.2. Experiment 2

**Figure 3:**
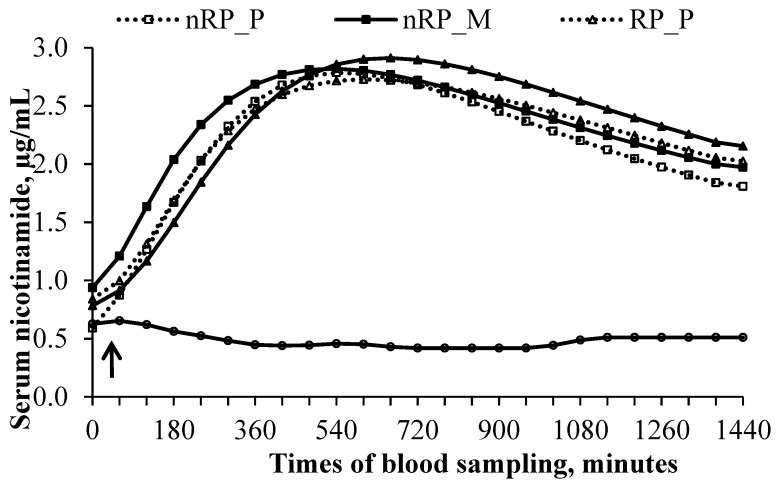
Mean serum concentration *versus* time curves of 24 g non-rumen protected (nRP) or rumen protected (RP) nicotinic acid in dairy cows following single *per os* administration oral dosage either in pelleted (P) or ground (M) concentrate form. The arrow marks the time of feeding the dietary treatments (Experiment 2).

Due to problems with venous catheter, not each cow received each dietary treatment. Four cows received CON, RP_P and RP_M, whereas three cows received nRP_P and nRP_M. It was ensured that no cow received the same dietary treatment twice. The daily intake of NA was 22.3 g NA and therefore slightly lower than the aimed 24 g. Changes in the niacin concentrations and in the pharmacokinetic parameters before and after oral dosing are illustrated in Figure 3 and listed in Table 3.

**Table 3:** Pharmacokinetic parameters of serum nicotinamide following administration of a single oral dose of 24 g non-rumen protected or rumen protected nicotinic acid either varying in dietary concentrate form (pelleted or ground) (Experiment 2).

Parameter	Unit	Dietary Treatment				*p-*Value		
		nRP_P ^1^	nRP_M ^2^	RP_P ^3^	RP_M ^4^	C ^5^	F ^6^	C × F
*B* ^**7**^	µg/mL	0.5 ± 0.2	0.9 ± 0.2	0.8 ± 0.2	0.8 ± 0.2	0.41	0.59	0.28
*k*_0.5_^**8**^	h	7.2 ± 1.1	4.3 ± 0.9	4.8 ± 0.8	6.4 ± 0.8	0.45	0.85	0.02
*R*_max_^**9**^	µg/mL	5.5 ± 0.5	3.9 ± 0.4	3.3 ± 0.4	4.1 ± 0.4	0.33	0.03	0.01
*K*_s_^**10**^	h	14.1 ± 3.0	19.5 ± 2.6	23.8 ± 2.3	21.0 ± 2.3	0.62	0.05	0.14
*I*_max_^**11**^	h	9.8 ± 0.9	8.8 ± 0.8	10.4 ± 0.7	11.5 ± 0.7	0.94	0.05	0.16
*AUC*_0–6_^**12**^	µg/mL	8.7 ± 1.0	10.7 ± 0.9	9.1 ± 0.8	8.3 ± 0.8	0.53	0.30	0.15
*AUC*_6–12_^**13**^	µg/mL	16.3 ± 0.6	16.7 ± 0.6	15.9 ± 0.5	16.5 ± 0.5	0.40	0.64	0.86
*AUC*_12–24_^**14**^	µg/mL	27.4 ± 1.8	28.3 ± 1.5	28.8 ± 1.3	30.8 ± 1.3	0.31	0.19	0.69
*AUC*_24_^**15**^	µg/mL	52.1 ± 2.1	55.6 ± 1.9	53.9 ± 1.7	55.7 ± 1.7	0.17	0.63	0.65

**^1^** nRP_P, cows received non-rumen protected (nRP) nicotinic acid (NA) in pelleted concentrate (P). **^2^** nRP_M, cows received nRP NA in ground concentrate (M); **^3^** RP_P, cows received RP NA in pelleted concentrate (P). **^4^** RP_M, cows received RP NA in ground concentrate (M); **^5^** C, dietary concentrate (pelleted (P) or ground (M)); **^6^** F, form of the NA supplement (non-rumen protected (nRP) or rumen protected (RP)); **^7^** Serum nicotinamide concentration at the beginning of the experiment; **^8^** k_05_, time for ½ R*max*; **^9^** the maximum theoretical nicotinamide concentration; **^10^** time indicative for the decreasing part of R*max*; **^11^** the time corresponding to R*max*; **^12^** Area under the curve 0 until 6 h after dosing; **^13^** Area under the curve 6 until 12 h after dosing; **^14^** Area under the curve 12 until 24 h after dosing; **^15^** Area under the curve after 24 h.

Thirty min before oral dosing, NAM concentrations of nRP_P, nRP_M, RP_P, and RP_M were 0.5 ± 0.2 µg/mL, 0.9 ± 0.2 µg/mL, 0.8 ± 0.2 µg/mL, and 0.8 ± 0.2 µg/mL and did not differ between the dietary treatments. The supplementation of nRP NA resulted in higher *R*_max_ values compared to RP NA (*p* = 0.03). Feeding nRP NA accelerate the rate of absorption (*p* = 0.05), because *R*_max_ of nRP NA occurred 1.7 h before the *R*_max_ of RP NA (*p* = 0.05). *K*_s_ was higher when supplementing RP NA compared to nRP NA (*p* = 0.05). The interaction between the form of concentrate and the galenic form of the supplement influenced *R*_max_ (*p* = 0.01) and *K*_0.5_ (*p* = 0.02). The use of nRP NA in pelleted form resulted in higher *R*_max_ and *K*_0.5_ values compared to the ground form, whereas in the case of RP NA, grounding of the concentrate provoked an increase of *R*_max_ and *K*_0.5_ values.

The relative bioavailability of nRP_P, RP_P, and RP_M compared to nRP_M were 95.2%, 94.3%, and 86.6%, respectively.

## 4. Discussion

### 4.1. General Remarks on Serum NAM and NA Levels in Bovine Blood

Literary statements regarding circulating blood levels of niacin and the presence of its vitamers are highly variable. As in the present study, Niehoff, *et al.* [12] (6 g) also detected only the vitamers NAM within the blood serum when using the HPLC-method. However, Campbell, *et al.* [21] and Morey, *et al.* [14] also used the HPLC method for the determination of blood niacin concentrations, but they detected both vitamers, NAM and NA. In this respect, it is important to point out that those research groups used bovine plasma as matrix. Therefore, observed differences in plasma and serum vitamer profile might be related to the use of different biological matrices. This might be confirmed by the results of Rungruang, *et al.* [22] who observed that the niacin concentration varied in different biological matrices with being highest in whole blood followed by red blood cells and leukocytes, milk, and plasma. Beside the explanation of different biological matrices as a cause for the absence of NA in the serum of the present animals, it can further be assumed that this might be due to a rapid conversion of NA to NAM. Ying [1] stated that mainly NAM is used as NAD precursor and therefore the conversion of dietary NA to NAM might be likely.

### 4.2. Effects of NA Supplementation on Serum NAM Concentrations

Present data reveal a dose-effect in experiment 1 with highest NAM concentrations when supplementing 24 g NA and lowest concentrations when supplementing 12 g NA. Present results disagrees with observations of Rungruang, *et al.* [22] who detected that plasma niacin concentrations increased linearly with increasing rumen protected niacin dose. A numerical and linear increase was obvious when supplementing nRP NA in the present study which was absent when supplementing RP NA. However, it remains fairly unknown why this dose-response relationship was not observed when supplementing RP NA. One explanation might be that these groups suffered from a reduced feed intake which cannot be more specified due to the absence of information of feed intake behavior. In interpreting the blood NAM levels of Experiment 1, it should be considered that just spot blood samples were examined. As Experiment 2 clearly showed that blood NAM levels depend on time relative to intake of NA supplemented feed it can well be that blood samples in Experiment 1 were collected at varying times within the expectable time-dependent systemic NAM fluctuations. However, this hypothesis for explanation of the failure of a clear dose-response relationship cannot further elaborated as standard deviations did not vary amongst all experimental groups.

In the study of Rungruang, *et al.* [22] plasma niacin concentrations were 1.23, 1.43, 1.50, and 1.65 µg/mL when supplementing 0, 4, 8, or 12 g to thermoneutral, lactating cows [22]. When using the linear regression model stated by Rungruang, *et al.* [22] the plasma niacin concentrations would increase up to 2.06 µg/mL when supplementing 24 g RP niacin to thermoneutral, lactating cows. Present results might confirm this prediction, as NAM concentrations ranged between 2.2 and 2.5 µg/mL at the beginning of experiment 1. Additionally, peak NAM concentrations were around 2.2 µg/mL in Experiment 2.

In contrast to the present study, where NA could not be detected, Rungruang, *et al.* [22] stated information about plasma niacin concentrations without differing between NA and NAM or other vitamers. As shown by Morey, *et al.* [14] NA accounts only for a very small fraction to total blood niacin concentrations which might be confirmed by the present results as NA was under the detection limit. Therefore, plasma niacin concentrations observed by Rungruang, *et al.* [22] might represent mainly the vitamer NAM making those plasma concentrations comparable to the present results.

Niehoff, *et al.* [5] and Hannah and Stern [23] suspect that there is a specific concentration of niacin within the rumen which regulates endogenous microbial niacin synthesis. This specific concentration might have regulatory functions like the inhibition of endogenous niacin synthesis in times of additional niacin supply associated with increased microbial degradation of the oversupplied niacin and the increased synthesis of niacin by ruminal microorganisms in the absence of niacin supplementation. Despite the continuous supplementation with 24 g nRP or RP NA, present NAM concentrations decreased towards the end of the experiment which might be a result of exceeding this specific ruminal concentration [5,23]. However, results of Jaster, *et al.* [24] might weaken the assumption of a specific ruminal concentration, because those researchers observed that serum NA concentrations increased continuously in *postpartum* dairy cows when supplementing 12 g niacin. On the other hand, the decrease in NAM concentrations might be related to a decreasing DMI, because feedstuffs used in ruminant nutrition are potential sources of niacin [18]. One explanation for the decreased DMI might be that the cows were nearly at the end of gestation which is often associated with a slight decrease in DMI. However, precise information on the DMI of the cows of the present study is lacking, because the consumption of the PMR was recorded group-specific. In addition, present results indicate that NAM concentrations decrease also when using encapsulated NA although this decrease was not as pronounced as in the case of nRP NA. If this decline in RP NA is due to the fact that the ruminal stability of this product is not as high as declared, remains unknown.

Therefore, present results highlight the need for future research aiming to increase the knowledge of relationships between oral niacin supply, feed intake, and ruminal metabolism.

The present results of experiment 2 indicated that maximum theoretical NAM concentrations were higher after nRP NA supplementation. However, as the *AUC*_24_ did not differ between nRP and RP NA, a lower bioavailability in RP NA as a consequence of an incomplete release of RP NA can be excluded within the observation period of 24 h. It should be noticed that NAM concentrations detected after 24 h were higher than the baseline level before feeding the supplements. Thus, the relative bioavailability reported herein applies only for the kinetic processes within the first 24 h. For comparative purposes between treatments these limitations have to be considered. However, one has to keep in mind that nRP NA reached maximum theoretical NAM concentrations 1.7 h earlier than RP NA, but that *K*_0.5_ values were higher for RP NA. The slower release of niacin from RP NA into the circulation combined with a longer persistence resulted in a similar *AUC*_24_ of nRP and RP NA although lower maximum theoretical NAM concentrations when supplementing RP NA. The present observations might confirm the statement mentioned above that both forms are able to affect serum NAM concentrations similarly. However, present results indicate different kinetic parameters which suggest slight differences in the degree of release from the matrix (liberation), absorption, degradation within the rumen, intestinal absorption, and/or elimination.

An important finding of experiment 2 is that serum NAM concentrations of both forms started to increase already 30 min after feeding. Recent studies showed that niacin is not able to accelerate liquid or particulate ruminal fractional turnover rate [6,7]. Mambrini and Peyraud [25] stated that the mean retention time (MRT) for ruminal liquids is 8.7 h and that the MRT increase with increasing particle size. It can be assumed that small amounts of ruminal fluid reach the duodenum within one hour. On the other hand, present results provide evidence that some part of absorption of both galenic forms might occur before the duodenum. However, to our knowledge, no study exists which focuses on ruminal absorption mechanisms of niacin. Nicotinic acid is a monocarboxylic acid and it was shown that NA is transported by a sodium-coupled monocarboxylate transporter (SLC5A8) in a mouse model [26]. By investigating the transcriptome of the rumen epithelium, Baldwin, *et al.* [27] detected that SCL5A8 is also present in rumen epithelium which provide indications for ruminal absorption mechanisms. Furthermore, there are indications that NA is transported by the monocarboxylate transporter-1 (MCT1) [28]. As MCT1 also exists within the rumen epithelium [29], ruminal absorption of NA seems likely.

### 4.3. Effects on Milk Production

Consistent with our results, Niehoff, *et al.* [12], Morey, *et al.* [14] and Aschemann, *et al.* [30], found no effect of NA on milk yield, FCM as well as milk fat, milk protein, and milk lactose yield. It may be concluded that the experimental time of the present study was too short to induce differences in milk performance, although NAM concentrations were moderately elevated. Another explanation is that present cows were in positive energy balance and as NA is able to balance enhanced lipid mobilization by the downregulation of lipolysis [13], it can be concluded that the absence of effects on milk production parameters is due to absent disturbances in energy metabolism.

## 5. Conclusions

In all, both forms are readily available for ruminant’s metabolism and are able to substantially affect serum NAM concentrations indicating that the ruminal degradation of nRP is not as massive as assumed formerly [4]. However, both experiments revealed significant protective effects of the encapsulated NA supplement as indicated by significantly higher blood levels under *ad libitum* conditions (Exp. 1), in a decelerated absorption of NA (significantly prolonged *I*_max_) and a significantly prolonged elimination from the systemic circulation (longer K_s_) possibly attributable to the slower release from the NA formulation (Exp. 3). Therefore, results of both experiments support the view that rumen protection of NA increases the systemic NA availability.

However, present results suggest that the absorption of oral supplemented nicotinic acid might have already start within the rumen. Therefore, investigations concerning absorption mechanisms should be followed up to increase the knowledge of niacin within the cow. The nature of the time-dependent decrease in blood niacin concentrations as well as a potential interaction with ruminal microorganisms needs to be elucidated in future studies where the level of total dry matter intake should be especially addressed as a source of variation.
